# Applying arterial enhancement fraction (AEF) texture features to predict the tumor response in hepatocellular carcinoma (HCC) treated with Transarterial chemoembolization (TACE)

**DOI:** 10.1186/s40644-021-00418-2

**Published:** 2021-08-12

**Authors:** Xiaonan Mao, Yan Guo, Feng Wen, Hongyuan Liang, Wei Sun, Zaiming Lu

**Affiliations:** 1 Department of Radiology, ShengJing hospital of China Medical University, 12# floor at 1# building, 39 Huaxiang Road, Shenyang City, 110000 Liaoning Province China; 2 GE Healthcare (China), Shanghai, China

**Keywords:** AEF, Texture analysis, Heterogeneity, Tumor response, TACE, HCC

## Abstract

**Background:**

To evaluate the application of Arterial Enhancement Fraction (AEF) texture features in predicting the tumor response in Hepatocellular Carcinoma (HCC) treated with Transarterial Chemoembolization (TACE) by means of texture analysis.

**Methods:**

HCC patients treated with TACE in Shengjing Hospital of China Medical University from June 2018 to December 2019 were retrospectively enrolled in this study. Pre-TACE Contrast Enhanced Computed Tomography (CECT) and imaging follow-up within 6 months were both acquired. The tumor responses were categorized according to the modified RECIST (mRECIST) criteria. Based on the CECT images, Region of Interest (ROI) of HCC lesion was drawn, the AEF calculation and texture analysis upon AEF values in the ROI were performed using CT-Kinetics (C.K., GE Healthcare, China). A total of 32 AEF texture features were extracted and compared between different tumor response groups. Multi-variate logistic regression was performed using certain AEF features to build the differential models to predict the tumor response. The Receiver Operator Characteristic (ROC) analysis was implemented to assess the discriminative performance of these models.

**Results:**

Forty-five patients were finally enrolled in the study. Eight AEF texture features showed significant distinction between Improved and Un-improved patients (*p* < 0.05). In multi-variate logistic regression, 9 AEF texture features were applied into modeling to predict “Improved” outcome, and 4 AEF texture features were applied into modeling to predict “Un-worsened” outcome. The Area Under Curve (AUC), diagnostic accuracy, sensitivity, and specificity of the two models were 0.941, 0.911, 1.000, 0.826, and 0.824, 0.711, 0.581, 1.000, respectively.

**Conclusions:**

Certain AEF heterogeneous features of HCC could possibly be utilized to predict the tumor response to TACE treatment.

## Background

Through the means of chemoinfusion and embolization, transarterial chemoembolization (TACE) has become one of the standard therapeutic options in the treatment of hepatocellular carcinoma (HCC) [1–3]. However, despite the rapid development of medicines and interventional devices, unbenefited patients to TACE still exist. These patients have always presented a great challenge to interventional radiologists in terms of both time and cost. A scientific prediction of tumor response before TACE would be very helpful in the integrated management of HCC.

According to the current guidelines on HCC management, mRECIST criteria [4] is used to judge tumor response based on specific imaging findings. The typical imaging finding of HCC lies in the enhancement pattern of arterial wash-in and portal/venous wash-out [1, 2, 4]. When HCC develops, the portal supply decreases, while the arterial supply increases and becomes more and more predominant [5–7]. The unique changes in perfusion status of the disease enable the application of perfusion analysis in HCC. Currently, with the development of computer engineering, arterial enhancement fraction (AEF), which reflects the ratio of the arterial supply to the portal supply, can be derived from routine enhanced CT images by aligning and subtracting unenhanced images from arterial and portal images [8, 9]. This provides us with a new method of perfusion analysis without subjecting patients to extra scanning and radiation exposure.

Different from traditional imaging analyses, which usually view the tumor as a whole, texture analysis disassembles the whole tumor into a number of independent pixels. Each pixel’s color scales (Perfusion values) can then be converted into high-dimensional quantitative data [10–12]. By a series of statistical calculation, multiple microscopic texture features can be extracted and further investigated in combination with clinical or histological findings [13, 14]. With an interest in exploring this new technology, the present study used the method of texture analysis to evaluate the application of AEF in predicting tumor response in HCC treated with TACE.

## Methods

### Patient management

This retrospective study obtained the approval from the institutional ethics committee of our hospital before implementation. Patients were recruited among the HCC patients treated in Shengjing hospital from June 2018 to December 2019. The step-by-step inclusion process involved the following criteria: (1) A diagnosis of liver cancer; (2) Aged 30 ~ 90; (3) Nonpregnant (for female patients); (4) CECT was acquired; (5) HCC was defined; (6) TACE was performed; (7) Imaging follow-up was conducted. The exclusion criteria included: (1) Uncorrectable artifacts; (2) Unmatched planes between phases; (3) Multiple HCCs (> 5); (4) Tiny HCC (< 1 cm); (5) Portal vein tumor thrombosis (PVTT) or portal vein cavernous transformation (PVCT); (6) Visible arterial-vein shunt (AVS); (7) Request to quit the study by the patient. Age, gender, hepatitis type, alcoholic background and family history of HCC were recorded. Liver function, renal function, coagulation function, ammonia, and alpha-fetoprotein (AFP) were tested. The China Staging System [1], Barcelona Staging System [2], and Child-Pugh Scoring system [15, 16] were used for integrated assessment.

Contrast enhanced imaging was acquired through a follow-up appointment that occurred within 6 months after TACE. The follow-up images together with the pre-TACE images were reviewed by two interventional radiologists who were neither co-authors, study designers, nor participants and were kept blind to the purpose of this study. The two radiologists were professionals in the interventional oncology field and had at least 5 years of working experience in abdominal imaging. They were tasked with classifying the tumor response in compliance with the modified RECIST (mRECIST) criteria [4] and with categorizing the outcomes of patients as Complete Remission (CR: The disappearance of any intratumoral arterial enhancement in all target lesions), Partial Response (PR: A decrease of at least 30% in the sum of the diameters of viable target lesions), Progressive Disease (PD: An increase of at least 20% in the sum of the diameters of viable target lesions) and Stable Disease (SD: A status that fits in between PR and SD but not qualify for either one) respectively.

### Image processing

CT scans were performed on a 128 row multi-detector CT (iCT 256, Philips, the Netherlands) with the scanning parameters as follows: Tube voltage 100kVp; Automatic tube current modulation; Pitch 0.993; Rotation time 0.5 s; Collimation 128 × 0.635; FOV 350 × 350 mm; Pixel size 0.8 × 0.8 mm; Plane thickness 3 mm. The enhanced images were acquired at the specific time points after the bolus injection of contrast (Visipague 270, GE, Ireland): Arterial phase 23 s; Portal phase 45 s; Delay phase 120 s. The volume of contrast was calculated by 1.2 ml/kg on body weight. The injection rate was 4.5 ml/s, followed by a 20 ml saline flush.

CECT images of DICOM format were downloaded from the CT workstation and loaded into CT-Kinetics program (C.K., GE Healthcare, China). In order to overcome difficulties in controlling breath-holding in imaging patients, 3D non-rigid motion registration was applied to improve the possibility of a good match between images of different phases. The aorta was chosen as the input artery, while the portal vein was chosen as the input vein. The density-time curve was obtained using a dual maximum slope model [17, 18]. The colored AEF map was generated automatically based on a pixel-by-pixel calculation of CTa-CTu/CTp-CTu (CTu: unenhanced CT value, CTa: aterial CT value; CTp: portal CT value).

The Region of Interest (ROI) of HCC was manually delineated along the tumor outline on the largest axial plane. The two interventional radiologists conducted the ROI delineation, with a compromise agreement if any inconsistency existed. A total of 32 AEF texture features were extracted through automatic statistical calculations, including Intensity-Based Statistical (IBS) features (MinIntensity, MaxIntensity, MedianIntensity, MeanValue, StdDeviation, Variance, VolumeCount, VoxelValueSum, Range, MeanDeviation, RelativeDeviation), Intensity-Based Histogram (IBH) features (Skewness, Kurtosis, Uniformity, Energy, Entropy), Gray-Level Co-occurrence Matrix (GLCM) features (Inertia, Correlation, InverseDifferenceMoment, ClusterShade, ClusterProminence, HaralickCorrelation), and Gray-Level Run-Length Matrix (GLRLM) features (ShortRunEmphasis, LongRunEmphasis, GreyLevelNonuniformity, RunLengthNonuniformity, LowGreyLevelRunEmphasis, HighGreyLevelRunEmphasis, ShortRunLowGreyLevelEmphasis, ShortRunHighGreyLevelEmphasis, LongRunLowGreyLevelEmphasis, LongRunHighGreyLevelEmphasis).

### Data statistics

Each AEF texture group, which comprised all patients, was initially tested using the Kolmogorov-Smirnov Test, to judge whether they fit into a normal distribution. Then, comparisons of AEF texture features were made between groups of “Improved” (CR + PR) and “Un-improved” (SD + PD) patients, as well as between “Un-worsened” (CR + PR + SD) and “Worsened” (PD) patients. An independent sample t test or Mann-Whitney U test was used as appropriate for continuous variables, while a chi-squared test or Fisher’s exact test was used for categorical variables.

To reduce the dimensionality of texture features and avoid the risk of overfitting, the spearman’s rank correlation test was used to exclude the redundant features (correlation coefficient |r| ≥ 0.9). Afterwards, the Least Absolute Shrinkage and Selection Operator (LASSO) algorithm was performed to identify the most useful features, with penalty parameter tuning conducted by 5-fold cross-validation. A multi-variate logistic regression was performed using the remaining features to estimate an “Improve” or “Un-worsened” outcome. A Receiver Operator Characteristic (ROC) analysis was applied to assess the discriminative performance of the models, including the area under the curve (AUC), the diagnostic accuracy, the sensitivity, and the specificity. A Calibration Curves Analysis (CCA) and a Decision Curve Analysis (DCA) were also applied, to assess the calibration degree of the models, and to evaluate their net benefit for clinical application at different probability threshold values.

All statistical analyses were performed with R (Version 3.5.1) and Python (Version 3.5.6). A two-tailed *p < 0.05* indicated statistical significance.

## Results

### Patient management

**Fig. 1:**
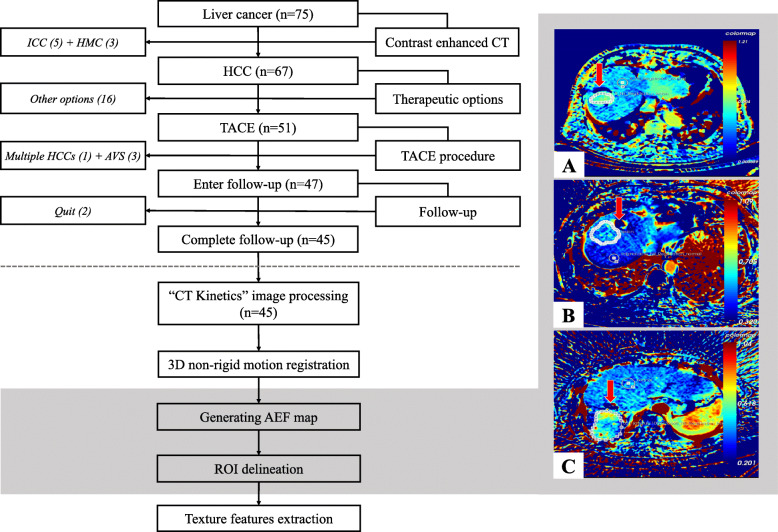
The workflow of the present study. HCC: Hepatocellular carcinoma; HMC: Hepatic metastatic cancer; ICC: Intrahepatic cholangiocarcinoma; TACE: Transarterial chemoembolization; AVS: Arterial-portal vein shunt; AEF: Arterial enhancement fraction; ROI: Region of interest. Numbers in gray represented the cases that passed every step through the inclusion process. Imaging processing was conducted by a CT kinetics program. Compared with CT images, AEF maps presented a better contrast of HCC (Warmer color) to the background tissue (**A, B** [19]**, C**). A non-perfusion area (Red arrows) indicated a good lipiodol accumulation from previous TACE intervention

Seventy-five patients with liver cancer were initially recruited for this study. Intrahepatic cholangiocarcinoma (ICC, *n* = 3), hepatic metastatic cancer (HMC, *n* = 5), and HCC (*n* = 67) were diagnosed, based on clinical and imaging findings. A small number of HCCs (*n* = 14) were further confirmed by histopathological findings. Fifty-one patients accepted TACE treatment for tumor control, as recommended by the Multiple Disciplinary Team (MDT) seminar. However, four patients were excluded after the operation due to the presence of multiple HCCs (*n* = 1) and visible AVS (*n* = 3) seen on angiography. After discharge, two other patients asked to quit the study for personal reasons. Eventually, forty-five patients completed the imaging follow-up and were ultimately enrolled (CR = 13, PR = 9, SD = 9, PD = 14). Their demographic and clinical characteristics are summarized in **(**Table 1**)**. A diagram is presented to illustrate the workflow of this study **(**Fig. 1**)**.

**Table 1:** Baseline of the enrolled cases

Characteristics	Improved	Un-improved	***p***	Un-worsened	Worsened	***p***
Gender	18^Male^/4^Female^	19^Male^/4^Female^	*0.945*	27^Male^/4^Female^	10^Male^/4^Female^	*0.203*
Age (years)	63.00 ± 9.18	60.09 ± 8.98	*0.288*	61.87 ± 9.14	60.71 ± 9.28	*0.698*
Weight (kg)	66.91 ± 11.98	70.74 ± 12.81	*0.307*	70.65 ± 11.87	64.93 ± 13.16	*0.155*
Hepatitis type	15^B^/7^Others^	19^B^/4^Others^	*0.260*	22^B^/9^Others^	12^B^/2^Others^	*0.287*
Alcoholic history	11^Yes^/11^No^	10^Yes^/13^No^	*0.661*	15^Yes^/16^No^	6^Yes^/8^No^	*0.731*
HCC family history	4^Yes^/18^No^	6^Yes^/17^No^	*0.524*	4^Yes^/27^No^	6^Yes^/8^No^	*0.025*
Tumor stage*	12^A^/7^B^/3^C^	8^A^/11^B^/4^C^	*0.596*	15^A^/12^B^/4^C^	5^A^/6^B^/3^C^	*0.343*
Child-Pugh score	5.55 ± 0.67	5.70 ± 0.88	*0.523*	5.61 ± 0.72	5.64 ± 0.93	*0.906*
Ammonia (mmol/L)	56.67 ± 18.44	73.35 ± 18.68	*0.004*	62.86 ± 21.16	70.36 ± 17.49	*0.223*
Albumin (g/L)	37.42 ± 6.06	35.98 ± 6.12	*0.431*	37.08 ± 5.63	35.80 ± 7.07	*0.517*
Total bilirubin (mmol/L)	21.43 ± 8.27	18.71 ± 1.30	*0.335*	21.38 ± 8.39	17.06 ± 10.93	*0.154*
Prothrombin time (S)	13.10 ± 1.63	12.63 ± 1.47	*0.315*	13.09 ± 1.55	12.34 ± 1.47	*0.137*
AFP (μg/L)	78.04^(2.10 ~ 11,041.00)^	26.05^(2.48 ~ 4466.00)^	*0.586*	78.04^(2.10 ~ 11,041.00)^	26.03^(2.48 ~ 4466.00)^	*0.364*

Note: Description of the baseline clinical data using Mean ± SD) for continuous variables that conform to normal distribution, Median^(Range)^ for continuous variables that don’t conform to normal distribution, and the actual number for categorical variables. Correspondingly, the statistical assessment applied Independent sample t test, Mann-Whitney U test, and Chi-squared test respectively. “*”: Tumor stages were judged based on Barcelona staging system

### Image processing

**Fig. 2:**
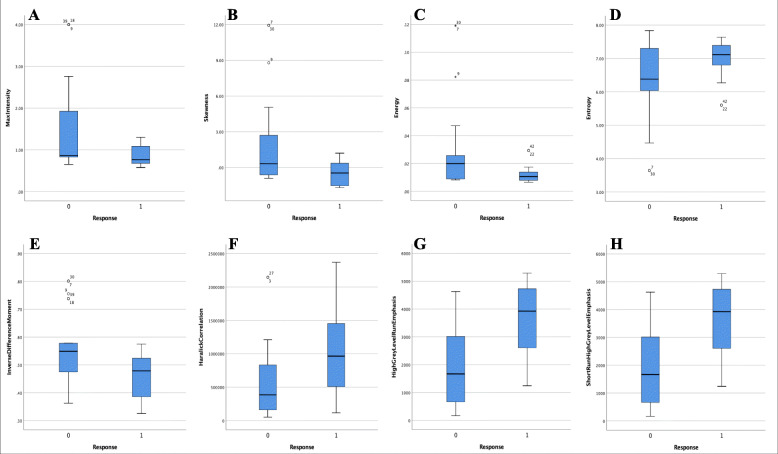
Box diagrams showing the difference of AEF texture features (*p* < 0.05) between “Improve” and “Un-improved” patients (Group 0: Un-improved; Group 1: Improved). Based on the definition of each texture, HCCs in “Improved” patients seems to produce more heterogeneous AEF values (**C:** Lower Energy; **D:** Higher Entropy; **E:** Lower InverseDifferenceMoment; **H:** Higher ShortRunHighGreyLevelEmphasis) and more left-skewed AEF values (**B:** Lower Skewness; **G:** Higher HighGreyLevelRunEmphasis), which indicates a more extensive and active angiogenesis, or arterialization, in the tumor

CECT scans took about 5 min on average with no accident occurrence. Neither immediate nor late contrast-related complications arose. C.K. image processing was conducted successfully in all patients. Forty-five HCC lesions were processed, according to the calculation of the largest tumor area by means of multiplying the pixel count and pixel size, the tumor sizes were: (1) Range: 1.25cm^2^ ~ 73.29 cm^2^; (2) Mean ± SD: 10.69 ± 1.89 cm^2^; (3) 95% CI: 6.70cm^2^ ~ 14.34 cm^2^. The AEF map showed a good contrast of HCC to normal liver parenchyma, illustrating an elevated proportion of artery/portal perfusion **(**Fig. 1**)**. Significant differences were found in 8 AEF texture features between “Improved” and “Un-improved” patients **(**Table 2**)**, while none were observed between “Un-worsened” and “Worsened” patients. Box diagrams of these features were plotted to illustrate their mathematical distribution **(**Fig. 2**)**.

**Table 2:** Comparisons of AEF texture features between “Improved” and “Un-improved” patients

AEF texture features	Improved	Un-improved	U	***p***
MaxIntensity	17.50^0.85/0.76^	28.26^1.54/0.86^	132	*0.006*
Skewness	18.59^–0.43/−0.45^	27.22^1.95/0.33^	156	*0.028*
Energy	18.14^0.01/0.01^	27.65^0.03/0.02^	146	*0.015*
Entropy	27.00^6.98/7.12^	19.17^6.30/6.38^	165	*0.046*
InverseDifferenceMoment	18.23^0.46/0.48^	27.57^0.56/0.55^	148	*0.017*
HaralickCorrelation	29.05^1093970.86/965593.50^	17.22^598050.88/386531.00^	120	*0.003*
HighGreyLevelRunEmphasis	29.25^3683.28/3936.19^	16.90^2072.46/1683.97^	104	*0.001*
ShortRunHighGreyLevelEmphasis	29.68^3685.10/3926.28^	16.61^2033.09/1667.16^	106	*0.001*

Note: Considering there were 32 texture features overall, only the features with significant between-group distinctions were listed in this table. AEF refers to the Arterial enhancement fraction. Data was described using the format of “Rank Mean^Mean/Median^”. Rank Mean was used to perform the Mann-Whitney U test, Mean and Median were listed as the references to help indicate the distribution of the values

### Data statistics

**Fig. 3:**
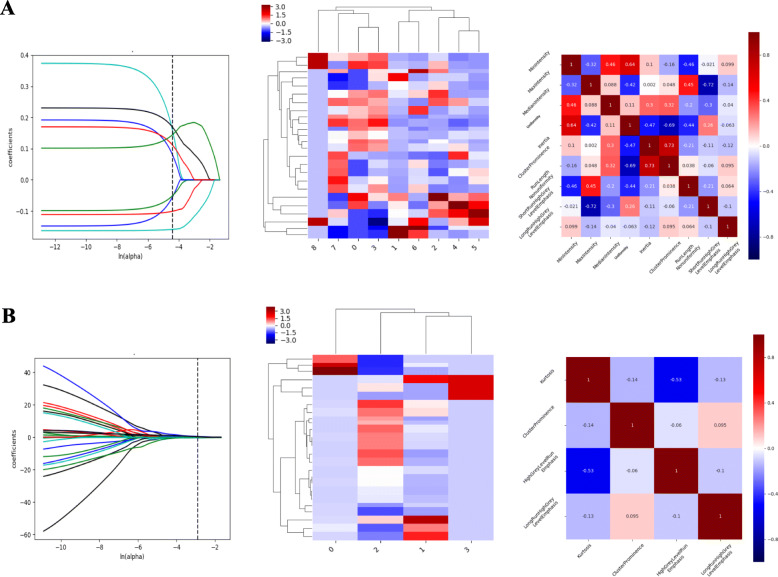
The remaining texture features were selected based on LASSO regression **(A1** and **B1)** which was used for a multi-variate logistic regression in “Improved” modeling (**A**) and “Un-worsened” modeling (**B**). **A2** and **B2** illustrated the clustering heatmap of the remaining features. **A3** and **B3** illustrated the correlation heatmap of the remaining features

As evidenced in the previous section, AEF texture features displayed significant dispersed distribution. Before conducting a logistic regression, all features were standardized to minimize this enormous dispersity. After the redundancy based on correlation analysis, 14 AEF textures were remained for subsequently analysis, including MinIntensity, MaxIntensity, MedianIntensity, Variance, Uniformity, Entropy, Inertia, ClusterShade, ClusterProminence, HaralickCorrelation, RunLengthNonuniformity, ShortRunLowGreyLevelEmphasis, ShortRunHighGreyLevelEmphasis, and LongRunHighGreyLevelEmphasis. After the LASSO regression analysis, 9 textures in modeling an “Improved” outcome and 4 textures in modeling “Un-worsened” outcome remained with non-zero coefficients **(**Fig. 3**)**.

**Fig. 4:**
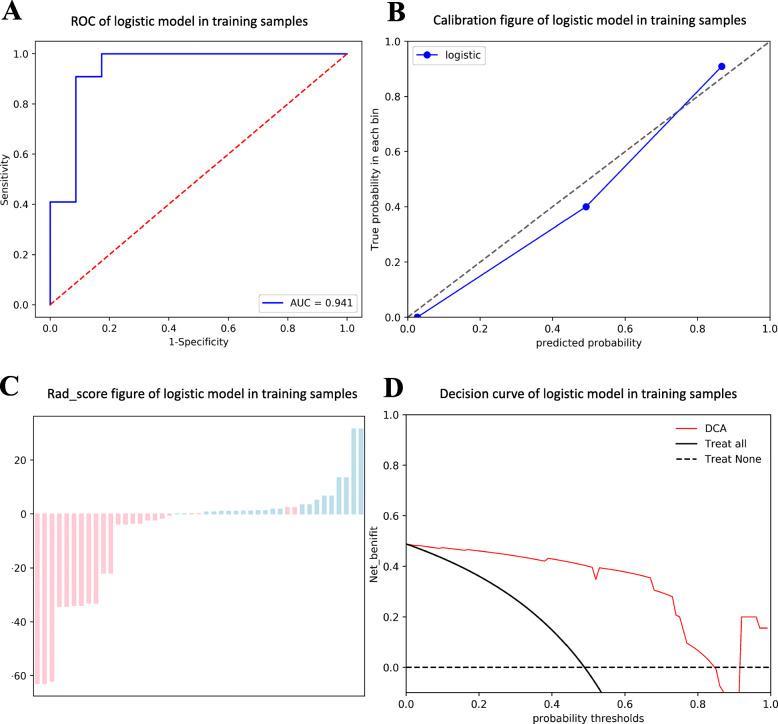
Receiver operator characteristic (ROC); Calibration curve analysis (CCA); Decision Curve Analysis (DCA). In predicting an “Improved” outcome, the ROC curve (**A**) shows an outstanding performance (AUC = 0.941). The CCA (**B**) shows a great consistency between the actual predicting performance (Solid blue line) and the ideal predicting performance (Dotted gray line). In the bar-chart (**C**), the horizontal level of “0” represents the best cut-off point of the model, bars above or below the “0” level respectively represent the two categories classified by the model (Red: Response; Blue: Non-response). The results in this study indicates a high predicting accuracy of 0.911 where only few mis-categorization (The red bars in the blue group) are observed. DCA (**D**) shows a coverage (Solid red line) of much more net benefit (y-axis) across the majority of the threshold probabilities (x-axis) in the model compared with the “treat-all strategy” (Solid black line) and the “treat-none strategy” (Dotted black line). This finding reveals the promising clinical usefulness of this model

**Fig. 5:**
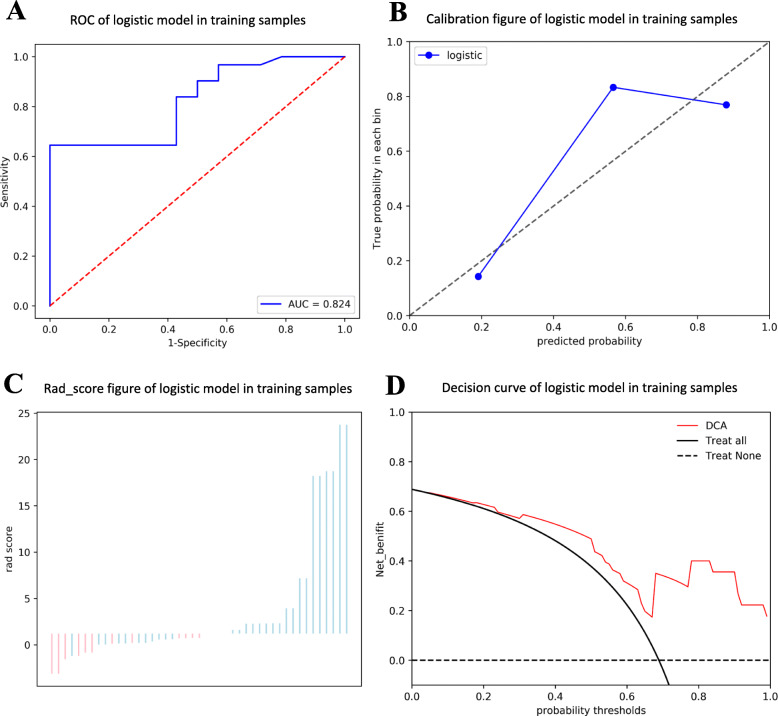
Receiver operator characteristic (ROC); Calibration curve analysis (CCA); Decision Curve Analysis (DCA). In predicting a “Un-worsened” outcome, the ROC curve (**A**) shows a good performance (AUC = 0.824). The CCA (**B**) shows a passable consistency between the actual predicting performance (Solid blue line) and the ideal predicting performance (Dotted gray line). The bar-chart (**C**) shows the best cut-off point of this model, which allows for a perfect prediction of a negative outcome (“Worsened”, Blue bar). Yet, the predicting performance for a “Un-worsened” outcome is unsatisfactory because of the presence of multiple false-positive cases, which results in a comprehensive predicting accuracy of 0.711. The DCA (**D**) shows the advantages of this model (Solid red line) compared with the “treat-none strategy” (Dotted black line), while its advantages over the “treat-all strategy” (Dotted black line) are not as significant

Finally, multi-variate logistic regression analyses were conducted using the remaining features. The formulas for modeling AEF in the prediction of “Improved” (Model A) and “Un-worsened” (Model B) outcomes are shown below with the coefficients listed in **(**Table 3**)**. The AUC, the diagnostic accuracy, the sensitivity, and the specificity of two models were 0.941, 0.911, 1.000, 0.826 and 0.824, 0.711, 0.581, 1.000, respectively. The prediction performance and the clinical usage of the models are shown in **(**Fig. 4**)** and **(**Fig. 5**)**, respectively.

$$ \mathrm{Model}\kern0.19em \mathrm{A}:\mathrm{fImproved}=-7.1555-8.5860\times \mathrm{MinIntensity}-17.1622\times \mathrm{MaxIntensity}+1.9920\times \mathrm{MedianIntensity}+12.5851\times \mathrm{Uniformity}+4.2988\times \mathrm{Inertia}+10.9129\times \mathrm{ClusterProminence}+0.5123\times \mathrm{RunLengthNonuniformity}-4.6584\times \mathrm{ShortRunHighGreyLevelEmphasis}-12.0606\times \mathrm{LongRunHighGreyLevelEmphasis} $$

$$ {\displaystyle \begin{array}{l}\mathrm{Model}\ \mathrm{B}:{\mathrm{f}}_{\mathrm{Un}\hbox{-} \mathrm{worsened}}=3.1287-0.5309\times \mathrm{Kutorsis}+1.8890\times \mathrm{ClusterProminence}+0.5523\times \\ {}\mathrm{HighGreyLevelEmphasis}+5.2633\times \mathrm{LongRunHighGreyLevelEmphasis}\end{array}} $$

**Table 3:** Coefficients of each AEF texture features in two prediction models by multi-variate logistic regression analyses

Model	AUC	Acc.	Sen.	Spe.	Items	Coef.	z	***p***
**A**	0.941	0.911	1.000	0.826	Intercept	−7.1555	−0.0000	*1.000*
					MinIntensity	−8.5860	−1.1469	*0.251*
					MaxIntensity	−17.1622	−1.1696	*0.242*
					MedianIntensity	1.9920	0.7683	*0.442*
					Uniformity	12.5851	1.1778	*0.239*
					Inertia	4.2988	1.2518	*0.211*
					ClusterProminence	10.9129	1.2056	*0.228*
					RunLengthNonuniformity	0.5123	0.3110	*0.756*
					ShortRunHighGreyLevelEmphasis	−4.6584	−1.0423	*0.297*
					LongRunHighGreyLevelEmphasis	−12.0604	−0.0000	*1.000*
**B**	0.824	0.711	0.581	1.000	Intercept	3.1287	0.0230	*0.982*
					Kurtosis	−0.5309	− 0.8292	*0.407*
					ClusterProminence	1.8890	1.7340	*0.083*
					HighGreyLevelRunEmphasis	0.5523	1.1414	*0.254*
					LongRunHighGreyLevelEmphasis	5.2633	0.0152	*0.988*

Note:Model A was applied for the prediction of “Improved” outcome; Model B was applied for the prediction of “Un-worsened” outcome. *AUC* Area Under Curve; *Acc*. Accuracy; *Sen*. Sensitivity; *Spe*. Specificity

## Discussion

After TACE intervention, an area with dense lipiodol accumulation is regarded as complete necrosis, where no enhancement is meant to be detected. If enhancement is detected, the remaining enhanced area will be judged residual [20, 21]. Although enhanced CT is the most commonly used imaging modality in follow-up [22] appointments, several studies have reported on CT’s limitations of which we should all be aware [23, 24]: (1) Routine enhanced CT can’t provide quantitative data [22]; (2) Lipiodol accumulation can cover or disturb the residual/recurrent enhancement inside/around the tumor [22, 25, 26]; (3) TACE brings the changes in tumor enhancement more often than the changes in tumor size [27]; (4) The devascularization effect of TACE can be heterogeneous and therefore difficult to manually measure [27]. Considering these shortages of routine enhanced CT, AEF can be an optional subject for investigation in the absence of AVS [8, 26, 28] with the following benefits: (1) AEF can improve the detection of HCC and can facilitate the detection of residual/recurrent lesions with faint arterial enhancement or slight wash-out in the portal phase [8, 9]; (2) AEF can successfully overcome the blight of already existing lipiodol because only the area with enhancement can be highlighted and picked out by subtraction [26]; (3) TACE eliminates the feeding artery of HCC so that the AEF of a tumor decreases while the AEF of liver tissue doesn’t [27]; (4) AEF reflects the status of angiogenesis, which is a vital appearance in tumor histology [29, 30]. These benefits enable us to correlate AEF quantification with survival data [27] and tumor response [31], and also to quantify clinical outcomes by comparing AEF before and after TACE treatment. The findings in other research or clinical trials indeed inspired us in considering the possibility of AEF being able to predict tumor response prior to the actual, invasive operation. This would allow for a comprehensive assessment of the necessity of TACE before it is conducted.

In the present study, we achieved a deeper exploration of AEF by applying texture analysis, which involved multiple detailed mathematical and spatial distribution features far beyond the comprehension of human eyes [32–34]. As introduced in the literature [35, 36], Energy and InverseDifferenceMoment are the textures that measure the homogeneity of an image while Entropy reflects local heterogeneity by specifying the uncertainty or randomness in the image values. Our results showed that those HCCs with a good response to TACE tended to reveal a bigger heterogeneity of AEF, this phenomenon can be explained by the pathophysiological process of HCC development. According to its definition, AEF is qualified to reflect the angiogenesis status of HCC [37–41], where the arterial and portal perfusion changes inversely rather than synchronously. HCC develops from the basic cirrhotic background or normal liver parenchyma where the portal perfusion should be predominant and almost even. The growth and maturation of HCC induces multifocal angiogenesis, more specifically called arterialization. Thus, during HCC development, the portion of arterial perfusion increases in more and more voxels, resulting in not only an elevation of AEF’s average, but also an enlargement of AEF variation among all the HCC voxels. Moreover, skewness measures the asymmetry of the distribution of values towards the Mean value [35, 36], depending on where the tail slants and where the mass of the distribution is concentrated. This mass of distribution can be positive (Right-skewed: Values more concentrated on the left side of the distribution curve, mean > median) or negative (Left-skewed: Values more concentrated on the right side of the distribution curve, mean < median) [42–44]. Among the other features we found distinctions in, HighGreyLevelRunEmphasis, measures the concentration of higher color scales, ShortRunHighGreyLevelEmphasis measures the joint distribution of shorter run lengths in the concentration of higher color scales [35, 36]. Our results indicated a trend of abundant, concentrated, but discontinuous arterialization in more voxels in “Improved” patients. In contrast, “Un-improved” patients revealed a status of continuously poor and scattered arterialization in more voxels. Therefore, we can infer that a lesion of HCC with a more heterogeneous and more left-skewed AEF correlates with a more extensive and active arterialization, which can enhance the tumor staining, facilitate the TACE procedure, and possibly amplify the effect of devascularization. Our personal experience also suggests that sufficient arterialization of HCC can strengthen the confidence in assigning TACE operation to patients and encourage better prognostic expectations.

It is believed that heterogeneity related texture features should be more or less correlated with the histological features of a tumor [45, 46] and, as assumed, be correlated with the biological behavior of a tumor that can influence prognosis. Thus, in order to discover more information about the relationship between AEF texture features and tumor response of HCC to TACE, we implemented a multi-variate logistic regression in which all the AEF textures were initially enrolled. However, consistent with other studies [34, 47, 48], it was necessary to apply some specific selection strategies to exclude redundant textures, in order to avoid exaggerating the importance of specific textures that had an interacting influence due to overlapping effect. In our research, two AEF based multi-texture models for predicting “Improved” and “Un-worsened” outcomes were successfully built, in which the IBS features, IBH features, GLCM features, and GLRLM features were all covered. These features all describe the mathematical distribution and spatial arrangement of AEF from different prospectives [35, 36]. The IBS and IBH features describe the distribution of pixel intensities within the image region defined by commonly used and basic metrics. The GLCM is a matrix that expresses how combinations of discretized grayscales of neighboring pixels. The GLRLM quantifies grayscale runs, which are defined as the length in number of consecutive pixels that have the same grayscale along a direction. To some extent, our AEF models, covering 4 classes of texture features, could be regarded as ones with comprehensive potential. Impressively, we gained an outstanding performance in encouraging diagnostic accuracy. The sensitivity for predicting “Improved” and the specificity for predicting “Un-worsened” outcome achieved the top value of 100%. In comparison, our results outperformed Zhao’s study [48], where she reported a combined nomogram for predicting early recurrence in HCC after partial hepatoectomy in training group, with a diagnostic performance (AUC = 0.878) higher than MRI radiomics model solo (AUC = 0.831) and clinicopathologic radiologic model solo (AUC = 0.797). We attribute the better performance of our AEF models to the application of perfusion processing, which allows non-invasive quantification of hemodynamic information [49, 50] that improves the detection [51, 52], grading [53–55], and monitoring [37, 56] of HCC. Furthermore, perfusion processing can also measure the viable vascular structure of HCC after treatments, which helps in tumor response assessment [57–60] and prediction [61–63], as well as in the prognosis and prediction of survival [64–66]. The novelty of the present study lies in a new methodology where texture analysis is conducted upon perfusion parameters, forming a combination of two sorts of functional imaging applications. Similar methodology was introduced in Liu’s study [47], but it was concerned with brain tumor (Pituitary macroadenoma). Kloth et al. [67] revealed the value of specific enhanced CT textures in predicting tumor response of HCC to DEB-TACE (Drug eluting beads TACE), where the perfusion parameters were applied, however, only to assess the tumor response, which means no perfusion texture features were extracted. Based on our investigation, studies combining perfusion quantification and texture analysis in HCC are still lacking.

Undoubtably, liver perfusion changes in a certain region when arterial perfusion is exceedingly increased due to highly active arterialization and when portal feeding declines significantly due to the presence of PVTT. However, in our opinion, these factors primarily influence the general perfusion features of liver tissue rather than the AEF texture features of HCC lesions. For example, the hypertrophic feeding artery indeed alters the blood flow velocity and volume (BF and BV) in the tumor. Consequently, the average AEF may also be elevated. However, what AEF texture features describe is the distribution of AEF values in a ROI, which quantifies the mathematical and spatial relationship among these AEF values. These values are neither the mean value nor the sum value. Histologically, the distribution features of AEF only result from differences in arterialization levels between any two adjacent tumor voxels or from differences in the position of a specific arterialization level. Thus, AEF texture features are theoretically decided by histological heterogeneity instead of by total arterial perfusion. On the other hand, HCC is fed basically by arterial neovascularization, especially in the late phase, where there is enough time to allow for significant tumor progression and sufficient arterialization. Therefore, late phase HCCs tend to present such abundant arterial feeding that portal feeding can be too insufficient to be observed. On an AEF map, tumorous tissue can be maximally highlighted due to an abundance of artery feeding. In contrast, although PVTT can block/restrict portal flow, healthy tissue can still be maximally hidden due to a lack of artery feeding. Thus, we do not believe that the presence of PVTT influences the AEF of HCC. Despite this, we do agree that the inherent microcirculation changes of a tumor may correlate with AEF features, which can be revealed by histology. Unfortunately, we did not acquire enough histological data in this study because: [1] According to the current guidelines, HCC can be diagnosed with adequate clinical data including disease history, imaging findings, tumor biomarkers, and so on. Thus, histological data is not necessary for the diagnosis of HCC [2]; Moreover, most participants in this study had been previously treated with multiple therapeutic means like TACE or radiofrequency ablation. These interventions typically produce a certain level of embolus deposition or necrosis inside the tumor, which can render new biopsies unreliable due to a possible false-negative result or inflammation infiltration.

There are several novel findings which are notable in this study: [1] An AEF map converted from CECT images may improve the viable tumor segmentation [2]; Like semi-functional imaging, AEF texture features can better reflect the pathophysiological status of a tumor [3]; The heterogeneity of AEF may imply the tumor response of HCC to TACE [4]; Certain AEF texture features may be able to influence and predict the tumor response of HCC to TACE [5]; Texture analysis on AEF may help in the selecting optimal HCC patients for TACE intervention. Concurrently, we are also aware of several limitations of this study, apart from the lack of histological data. First, a validation group was not set because of the limited sample size. Second, the lack of standardization in texture analysis is a concerned due to the diversity of imaging equipment, scanning protocols, processing software, and ROI segmentation [68–71]. Finally, we found AEF MaxIntensity and HaralickCorrelation showed between-group distinction, but we were not convinced that they are qualified in demonstrating tumor heterogeneity, because: [1] MaxIntensity represents the single pixel with the highest AEF value in the ROI, which is too independent and individualized to reflect the texture feature of the whole ROI; and, [2] HaralickCorrelation measures the linear dependency of a pixel to its neighboring pixels [72], which can have diverse values between certain adjacent pixels but presents the same overall value for the whole ROI. Therefore, further studies involving more cases and validation group should be encouraged, as more findings on the relationship between the thousands of perfusion texture features and the clinical outcomes of HCC are needed.

## Conclusion

By using perfusion conversion and texture analysis on CECT images, the ability of AEF to influence and predict tumor response in HCC to TACE was demonstrated. AEF can predict tumor response through certain texture features and through its heterogeneity. These findings can improve the selection process of TACE patients and contribute to more favorable outcomes in this intervention.

## Data Availability

The data that support the findings of this study are obtained from the Electrical Medical Record (EMR) and Picture Archiving and Commumication System (PACS) of Shengjing hospital, which involves a lot of private information of the participants and particular policies/regulations of Shengjing hospital. Strict restrictions apply to the availability of these data for public sharing. Additionally, the raw data also contain information that other ongoing study is using. Therefore, the raw data can’t be publicly shared at this time.

## Abbreviations

HCC: Hepatocellular carcinoma
TACE: Transarterial chemoembolization
AEF: Arterial enhancement fraction
CECT: Contrast enhanced computed tomography
mRECIST: Modified response evaluation criteria in solid tumours
ROC: Receiver operator characteristic
AUC: Area under curve
